# Pushing for change: a qualitative study of the experiences of elite athletes during pregnancy

**DOI:** 10.1136/bjsports-2021-104755

**Published:** 2022-02-08

**Authors:** Margie H Davenport, Autumn Nesdoly, Lauren Ray, Jane S Thornton, Rshmi Khurana, Tara-Leigh F McHugh

**Affiliations:** 1 Program for Pregnancy and Postpartum Health, Faculty of Kinesiology, Sport, and Recreation, Women and Children's Health Research Insitute, Alberta Diabetes Institute, University of Alberta, Edmonton, Alberta, Canada; 2 Faculty of Kinesiology, Sport, and Recreation, University of Alberta, Edmonton, Alberta, Canada; 3 Public Health and Family Medicine, University of Western Ontario Schulich School of Medicine and Dentistry, London, Ontario, Canada; 4 Departments of Medicine and Obstetrics and Gynecology, University of Alberta, Edmonton, Alberta, Canada

**Keywords:** athletes, pregnancy, sport

## Abstract

**Objectives:**

Athletes train and compete at the elite level during their reproductive years, yet sport policies that support pregnant athletes are lacking. The experiences of elite athletes during pregnancy are vastly under-represented, and such voices are needed to support evidence-informed policy. Thus, the purpose of this qualitative study was to describe the experiences of elite female athletes as they navigate pregnancy, and to identify sport policy considerations regarding participation during pregnancy.

**Methods:**

Twenty athletes (mean age 35±5 years) who, within the last 5 years, had trained or competed at the elite level immediately prior to becoming pregnant were included. Data were generated via one-on-one semistructured interviews that were audio-recorded, transcribed verbatim and analysed through a process of content analysis.

**Results:**

The findings of this study are represented by one overarching message: mother versus athlete, and five main themes: (1) pregnancy planning and fertility, (2) pregnancy disclosure and discrimination, (3) training pregnant athletic bodies, (4) safety concerns, and (5) supportive network and equitable funding.

**Conclusion:**

In-depth stories shared by participants highlight the many significant decisions athletes must make as they navigate pregnancy alongside elite sport participation. The shared experiences of pregnant athletes highlight clear challenges that should be considered in the development of sport policy and practices that are inclusive and supportive of female athletes.

## Introduction

Over the last decade, elite athletes have begun to challenge stereotypes regarding participation in elite sport during pregnancy. From marathon runner Paula Radcliffe to tennis star Serena Williams, and most recently sprinter Allyson Felix, female athletes are pushing against the societal narrative that they should ‘take it easy’ during pregnancy. Although extensive literature supports the safety and benefits of prenatal exercise, these data are primarily limited to the general obstetric population. Current exercise guidelines outline 150 min of moderate-intensity physical activity each week.^1–6^ However, these conservative recommendations do not serve elite athletes who substantially exceed this advice during training. Several studies demonstrate that sport and exercise participation declines in pregnant athletes due, at least in part, to a lack of research and policy in this area.^7 8^

In 2016, the International Olympic Committee (IOC) convened a meeting of experts in prenatal exercise and produced five documents reviewing available literature regarding pregnant athletes.^9–13^ At that time, there was minimal information regarding the safety and benefits of high-intensity, long-duration or extreme volumes of exercise. More recent work suggests elite sport participation during pregnancy is not associated with adverse events during pregnancy, labour or delivery.^14 15^ Yet from a cultural standpoint, pregnant athletes are often viewed negatively by observers out of fear that the fetus is placed at risk when a pregnant athlete participates in sport.^16^ This is compounded by the media, which have played a role in stigmatising pregnant athletes, even as our view of exercise in pregnancy shifts.^17^

As more elite female athletes train and compete during their reproductive years, it is critical that policies to support the well-being of athletes also evolve. Over the last 5 years, there have been calls to action by international (ie, the IOC) and national organisations (ie, Canadian Society for Exercise Physiology) to conduct research with this overlooked group of athletes. However, the experiences of elite female athletes are vastly under-represented. The few studies highlighting the experiences of pregnant or parenting elite female athletes document the uncertainty and stress they face within their careers,^18^ and how athletes must learn to adopt new roles as ‘mother–woman–sportswoman’.^19^ Also, through textual analysis, researchers^17^ have examined the cultural and societal complexities faced by pregnant elite athletes. Research involving elite female athletes is necessary to support evidence-informed policy regarding sport participation in pregnancy. Thus, the purpose of this qualitative study was to define the experiences of elite female athletes as they navigate pregnancy and to outline sport policy supporting elite sport participation during pregnancy.

## Methods and analysis

We employed a qualitative study design as it is often used when there is a dearth of knowledge within a subject area and when a detailed description is sought.^20^ Between January and June 2021, we recruited 20 elite athletes through social media (ie, Twitter, Facebook and Instagram) and word of mouth via a purposeful and snowball sampling approach.^21^ To be eligible, athletes had to be ≥18 years old and to have trained and/or competed at the highest level of their sport immediately prior to and during pregnancy within the last 5 years (2016–2021). Prior to participation, individuals provided written informed consent, and completed a brief questionnaire about their sporting background, demographics and an overview of their pregnancy experiences. The questionnaire included items such as the highest level of competition achieved, the duration/type of training during pregnancy and specifics of delivery (eg, birth weight). Once eligibility was confirmed, participants engaged in a 1-hour one-on-one semistructured interview; the interview guide was informed by sport and pregnancy-related literature and a research advisory board (RAB). The interview guide consisted of 12 main questions relating to athletes’ experiences, and the semistructured nature of the guide allowed the interviewers the flexibility to ask participants to elaborate on specific aspects of their responses. All interviews were conducted by video conferencing and lasted an average of 60 min. Interviews were audio-recorded and transcribed verbatim by Otter.ai. More detailed methodology and analysis are available in the online supplemental material. The public was not involved in the design of this study.

10.1136/bjsports-2021-104755.supp1 Supplementary data

### Data analysis

Consistent with a qualitative description study design, a three-phase process of content analysis (ie, preparation, organisation and reporting), as outlined by Elo and Kyngäs, was used to analyse the data. In terms of the preparation phase, interviews were audio-recorded, transcribed verbatim and checked for accuracy.^22^ Immediately following each interview, a narrative summary of each interview was created by the interviewer and shared with members of the RAB. To familiarise themselves with the data, the RAB read and reread the transcripts and narrative summaries. As well, during the preparation phase of analysis, groups of words were identified as the unit of analysis. The organisation phase involved developing a codebook and identifying possible categories, patterns and themes. Interviewers (AN and LR) led the process of open coding and, during weekly team meetings from January to June 2021, the RAB discussed codes, patterns and the identification of themes. The data were classified and reclassified into comprehensive higher-order themes until consensus was achieved among the RAB. Results were then shared with all participants, who were asked to provide feedback or comments to be integrated into the reporting of findings. During the reporting phase of analysis, direct quotes from participants were used to describe and support each theme.

## Results

Twenty-four athletes responded to our request for participants. Two declined to participate and two did not meet our inclusion criteria. The experiences of 20 athletes from 11 Olympic (team and individual) sports are represented in this study. Most of the participants were from North America with additional athletes from Europe. Participants were an average of 35±5 years of age with 15 of 20 currently training or competing at the elite level (see table 1). To ensure anonymity of the participants, a complete list of the athletes’ sports is not provided, and pseudonyms have been used in the reporting of the results.

**Table 1:** Participant characteristics

Age (years)	35±5
Retired	20% (n=4)
Elite sport participation (years)	8.8±5.7
Type of sport	
Team	50% (n=10)
Individual	50% (n=10)
Highest level of competition	
Olympic Games or world championships	50% (n=10)
National/international competition	50% (n=10)

The findings of this study are represented by one overarching message: mother versus athlete, and five main themes: (1) pregnancy planning and fertility, (2) pregnancy disclosure and discrimination, (3) training pregnant athletic bodies, (4) safety concerns, and (5) supportive network and equitable funding. It is important to note that although the themes are presented separately, elite female athletes’ experiences of pregnancy are complex and therefore the themes are not mutually exclusive. Themes are described and supported by direct quotes from participants.

### Mother versus athlete

This overarching message represents a common and notable thread reflected in the shared stories described across all five main themes (see table 2). It is presented as an overarching message to emphasise the many significant decisions participants encounter as they navigate pregnancy alongside being an elite athlete. Participants described how there is a common societal narrative to suggest they must choose between being an athlete or becoming pregnant. On becoming pregnant while training at an elite level, athletes said they received messages such as ‘happy retirement’ or ‘congratulations you’re done (competing) now’. As such, athletes found themselves questioning what was possible. ‘Nicola’ said, ‘…do I compete at Nationals or do I try and get pregnant? Because I can't do both’.

**Table 2:** Actionable steps to support elite female athletes

Themes	Example supporting quotes	Policy recommendations and actionable steps to support pregnant elite athletes
Pregnancy planning and fertility	‘We had tried to get pregnant, and we weren't successful. We had a very specific timeline’. (Jillian) ‘I wasn't having a regular menstrual cycle because of heavy training volumes, I have a leaner build. So, it was a lot harder for me to get pregnant the first time around’. (Amy)	Educate coaches, medical professionals and athletes of reproductive age about the impact of training on the menstrual cycle and fertility. Develop clear maternity leave policies and support to allow athletes to plan for pregnancy.
Pregnancy disclosure and discrimination	‘I wouldn't discuss it with them [coaches] at all until I knew until I had to… I was worried that they would question my commitment’. (Leslie) ‘I think that more than anything, it was my own insecurities of thinking that as a female, if I get pregnant, they're not going to accept me anymore’. (Emma)	Provide training sessions to athletes, coaches, medical professionals and sporting organisations on promoting a supportive environment for pregnant athletes. Increase the visibility of athletes who have successfully navigated pregnancy and elite sport. Develop antidiscrimination laws to support athletes who become pregnant.
Training pregnant athletic bodies	‘It was difficult to accept early on… I sort of saw my [training] plummet … I did have quite a hard time with reconciling in my own head that I was in a different place and that I was going to have to back off’. (Pamela) ‘There may be guidelines that exist, but I'm not convinced that they apply to athletes’. (Jillian)	Increase high-quality research into the impact of elite-level training during pregnancy (eg, fertility, safety, limits and health outcomes). Improve knowledge translation of evidence-based recommendations to athletes, coaches and healthcare providers.
Safety concerns	‘I got pregnant while I was competing and pulled out of a race because I thought I was pregnant. And just thought, ‘Oh my gosh, I don't want to be like damaging the fetus’’. (Stella) ‘I want there to be more concrete answers about what I can and can’t do … can I actually harm the fetus … if it’s going to harm the baby I’m not going to do it?’ (Mallory)	Increase high-quality research into the impact of elite-level training during pregnancy. Provide training and education to healthcare providers on evidence-based recommendations for training and competing during pregnancy.
Supportive network and equitable funding	‘A supportive partner has been huge for me, a supportive coach, someone who can understand the woman’s body and what it goes through [during pregnancy]’. (Jana) ‘I don't think it’s very convenient for anybody when an elite woman becomes pregnant. But what is a support right now is more social media attention for pregnant athletes and more shaming of sponsors that don't treat their pregnant athletes or postpartum athletes properly, and the rewriting of a lot of sponsorship agreements’. (Cassidy)	Normalise and value pregnancy and elite sport by developing best-practice policies and funding to support pregnancy. Promote and provide greater visibility of athletes who successfully navigated pregnancy and elite sport. Develop networks for athletes considering pregnancy to speak with others about shared experiences.

Participants explained how becoming pregnant means they must make challenging decisions regarding taking time off from training and competing. As ‘Emma’ explained, ‘There’s never a good time to, or it feels like there’s never an appropriate time, to be able to take that time off’. Similarly, ‘Leslie’ shared, ‘If you want to take any time off, someone else will probably get your spot. You might lose your funding; you might not be competitive when you come back. So it’s very risky’.

Recognising all athletes may face choices about becoming a parent, ‘Joelle’ explained how such ‘choices are harder’ for women who have ‘…childbearing responsibility, if that’s what you choose to do, we have to make some tough decisions of when we're going to do it, and how it’s going to affect our lives and our sport and our career’. Participants also explained how there are already gender inequalities in sport, and having to make decisions regarding pregnancy, or returning to sport after pregnancy, is added pressure for many athletes. As stated by ‘Tamera’,

I listened to a great podcast of a girl who got pregnant…how she had to return to competitive sport six weeks after having a baby. She’s not allowed to have a pregnant body. You're not allowed to have a pregnant body in sport, if you're an elite athlete.

Given the difficult decisions many athletes need to make regarding pregnancy and being an elite athlete, participants described the importance of representation and support from other pregnant elite athletes. As ‘Robyn’ explained, ‘I know there are books out there, but hearing it from other mothers who just went through it, or tips that they learned, would be helpful’.

### Pregnancy planning and fertility

Planning for pregnancy, especially when considering potential fertility challenges, was identified as a key concern for many of the participants. Participants explained how competition schedule, funding and potential fertility issues are all important to consider when trying to conceive as an elite athlete. As ‘Heidi’ explained, ‘During an Olympic cycle, you want to get pregnant in the first year of the cycle before your quadrennial…like you have a very narrow window to try and succeed or wait another four years’. Despite efforts to plan pregnancy, participants explained how becoming pregnant was not always in their control and did not necessarily work out as planned.

Athletes also highlighted the importance of considering factors such as funding and sponsorship when planning for pregnancy. As ‘Amy’ suggested, ‘The only tricky part is you have to fall into that timeline to get the funding, which makes it stressful for somebody, if it’s taking longer to get pregnant’. Similarly, as stated by ‘Robyn’, ‘The way that the contracts work with sponsors is that all of your bonuses are based on performance, right?…they rate you for the next year, and your contract is based on how you compete’. Participants explained how these financial implications greatly impact their decisions regarding being pregnant as an elite athlete.

In addition to funding implications, participants explained how fertility was another important consideration in planning pregnancy. Training was described by some participants as negatively impacting the viability of conception, and ‘Tamara’ questioned whether her level of training impacted her fertility. She explained,

I don't think they knew why I couldn't get pregnant. I think sometimes they just don't know, right? It’s complicated. But I felt like I knew… Lots of women can get pregnant at that level of training, and there are also lots of women that can't.

Scaling back on training volume to increase the chances of conception was described by some of the athletes who identified themselves as having ‘leaner bodies’ or ‘irregular menstrual cycles’. However, participants also explained how they felt conflicted when reducing training because this had a negative impact on their sport performance.

Regardless of training level, athletes noted there are sports where their peak performance and fertility windows overlap, which can make it even more difficult to conceive. As described by ‘Darcy’, ‘You have this fertility window that is important… and we have a performance window where we can be optimal’. She explained how these ‘overlapping’ windows often require athletes to make critical decisions about whether they should compete, become pregnant or try to do both. Athletes also discussed how they may have missed the fertility window because of their training, and therefore required fertility treatment and desired some form of support. ‘Norah’ shared, ‘Like you gave your body to your country, maybe they can do something to help you along your way’.

### Pregnancy disclosure and discrimination

Athletes described their hesitation to disclose their pregnancy with coaches or their sport organisation, in fear of losing their position on their team or having their commitment to the sport questioned. As ‘Norah’ explained,

I feel like I can't have open communication [with coaches] because I'm so afraid of what will be taken from me. That’s not really fair…I'm also keeping my mouth shut just in order to preserve what’s mine type of thing.

Participants also described how they felt uncertain whether they would be accepted and respected after they disclosed their pregnancy. For example, ‘Kendra’ said, ‘I was a little bit nervous to share the news with them, just because I wasn't sure how they would react’. Despite such hesitancy, some participants explained why they disclosed their desire to become pregnant, even prior to becoming pregnant. For example, athletes described how they had to notify their sport organisations that they planned to undergo fertility treatments as they knew such treatment could interfere with antidoping policies. As well, ‘Mallory’ said, ‘The first person I told was my trainer, because I wasn’t ready to tell the team yet. But I felt like the trainer needed to know given my [lower] energy levels’.

While participants acknowledged they were hesitant to disclose their pregnancy, participants also explained how certain sports have managed to create a supportive environment for pregnant elite athletes. As such, pregnancy disclosure in such environments might not be as stressful. As ‘Kara’ explained, ‘There’s a lot of high-performance athletes that have been pregnant and delivered and played through pregnancy, come back postpartum and won championships. So there’s a lot of, I guess, powerful women in the sport’. Participants, such as Kara, shared stories to suggest disclosing pregnancy in some sports (but not all sports) has been made easier by other female athletes who have successfully navigated pregnancy as an elite athlete and serve as role models, leaders and trailblazers.

### Training pregnant athletic bodies

Participants described how they had personal expectations and external pressures regarding what they could and/or should do in terms of training. Often these expectations and pressures did not align with what was possible. For example, ‘Jana’ shared, ‘I expected that I would be able to [train] through my entire pregnancy. But what I learned quickly and had been told so many times is that pregnancy is different for every woman’. Participants described that when they became pregnant, they found it challenging to reconcile or accept they could not train or compete at prior levels. In describing how her training changed when she became pregnant, Emma said ‘…It was incredibly mentally frustrating’.

The athletes also highlighted how they felt ill-equipped to make the right decisions for their training while pregnant. As described by ‘Charlotte’, ‘Having been an athlete for so long, and knowing how to train, made it hard to all of the sudden not necessarily know how to train’. Many participants shared similar sentiments with Charlotte. For example, Emma shared her experience of training early in her pregnancy. She said, ‘It just hurt so bad. I think that was the first time in my training that my lack of knowledge really affected me, physically. I was rudely awakened to what I can do vs what I should do’.

Athletes described their scepticism towards the guidelines and training programmes provided by healthcare providers, trainers and coaches because of the lack of evidence-based information directly relevant to athletes. As ‘Nicola’ noted, ‘I was just doing my own thing, going off my own gut instinct. I don't find healthcare professionals get the athlete side of it’. Similarly, ‘Robyn’ said ‘The doctors don't know about training and pregnancy’. Recognising they had little guidance with respect to training during pregnancy, some of the athletes explained how they relied on their own research and information gathering skills to determine how to train while pregnant. For example, Emma explained, ‘I have a lot of distrust in some ways and skepticism…I don't trust my medical providers anymore, and I feel like I have to do all that research on my own’. In contrast, other athletes highlighted the importance of having a healthcare provider with expertise related to pregnant elite athletes. As stated by ‘Jillian’, ‘I looked to my pelvic physiotherapist for most guidelines because, to me, she was the expert in my core and my pelvic floor. And those were the things I was most concerned about’. Some of the participants described how they trusted pelvic floor specialists to provide them with modifications to exercises while pregnant.

Participants described how their healthcare providers, trainers and coaches trusted athletes would know how to train during pregnancy. ‘Joelle’ said, ‘My doctor knew that I was a competitive [athlete] and worked out three to four days a week and [they] said keep doing it as long as you feel good, and there’s no problem’. ‘Robyn’ also explained how her physician said that it was ‘Safe to do what you are used to, but dial it back. But, I don't think they understood quite the level of what I was used to doing’. This discrepancy between the trust placed in the athlete and the athletes’ distrust of healthcare providers led to further uncertainty about the safety of elite-level training during pregnancy.

### Safety concerns

Participants’ experiences of training while pregnant were layered with concerns of their chances of getting pregnant, and the potential impacts of training on themselves and their baby. While trying to get pregnant, ‘Nicola’ recalled ‘I was still training fully. And I was like, ‘Oh my God, can I go in a sauna? Oh, my God, should I be training?’ Maybe this is the reason I'm not getting pregnant’. Athletes also described an uncertainty around not knowing the consequences of maintaining their training during pregnancy. As stated by Jana, ‘I push my body to the limit right now because it’s only me I have to worry about, but when there’s another person inside of you that you're growing, you want to maintain it and be healthy’. Athletes often described searching for accurate information to determine the safety of elite-level training. As ‘Pamela’ stated, ‘Just sort of finding the right information in terms of what is safe, what is not safe. You know, are there certain things that I should be doing? Are there certain things that I shouldn't be doing?’ In a quest for such information, athletes such as ‘Tanis’ described how they found themselves ‘googling’ information to see what type of exercises would be safe for both themselves and their unborn child. However, athletes acknowledged the lack of evidence-based information regarding training during pregnancy among elite athletes.

### Supportive network and equitable funding

Having a supportive network was noted as a huge facilitator in maintaining a career as an elite athlete during pregnancy. Athletes discussed the importance of having their spouse, coach, family and teammates supporting their desire to compete and train as an elite-level pregnant athlete. As noted by ‘Joelle’, ‘I've got a very supportive husband and family, my in-laws and my family are very supportive. Especially now with a child, I'm going to rely on them even more’. Participants emphasised the need for coaches to be understanding and supportive of their desire to be a pregnant athlete. As stated by Leslie, ‘My coach was awesome…super supportive and would modify things…and didn't make me feel weird’. The athletes shared various stories that demonstrated that not all coaches are understanding of pregnancy. As stated by Jillian, ‘I think I'm done with male coaches, for now. If I can work with female coaches, I think I will try to. I'm not offended. There’s nothing wrong. I just don't feel understood [by male coaches]’. In addition to coaches, athletes explained how their teammates also played an important role in their supportive network. Pamela said, ‘One of the girls I [train] with the most often… we have a lot of conversations about that transition away from being an elite athlete…She’s someone that I can bounce those ideas off’. Similarly, ‘Kendra’ explained, ‘I think our team is really understanding that family kind of comes first’.

However, financial support during the prenatal and postnatal periods was also described by athletes as critical to their ability to remain as elite athletes. As described by Amy, ‘Being a female athlete and trying to make that your job is not easy. For me, personally, having the support of the government assistance funding, the [government] funding has been huge’. Participants also described how the government funding system has recently made some positive changes to support athletes who are pregnant. As Jana said, ‘Pregnancy used to be classified as an injury. And now it’s been classified as a pregnancy card. So, you can still receive your support through the period that you're pregnant and through delivery’. Similarly, Amy explained how within her sport organisation pregnant athletes can maintain their ‘carding status’ and continue to receive ‘carding money’. Despite some athletes feeling they were financially supported during pregnancy, others described the rules around funding are not clear, and often differs across, and even within, the various national sport organisations. For example, despite Amy describing she received adequate financial support, she noted other athletes in her organisation did not receive any support. The lack of secure funding for pregnant athletes was identified as a key reason why athletes were considering the financial implications of pregnancy and their careers. When they did not align, athletes chose to delay pregnancy or retired from sport.

## Discussion

For centuries, men have dominated elite sport; however, gender parity was nearly reached for the first time at the 2020 Summer Games in Tokyo. As more female athletes train and compete at the elite level during the reproductive years, it is critical sport policies evolve to support the health and well-being of all athletes. Our study of recently pregnant elite athletes identified an overarching theme, which represented the struggle between the new identity of becoming pregnant while maintaining their identity as an elite athlete. This unifying theme was present through each of the five main themes.

Previous research has also identified the struggle to manage two sometimes conflicting identities. Martinez-Pascual *et al* described how elite athletes felt their identity as a mother overtook their identity as a sportswoman and, as a result, many athletes described the guilt about trying to do both.^19^ Such identities are further compounded by the cultural and societal complexities of media portrayals of elite pregnant athletes.^17^ Most recently, Darroch *et al* identified the lack of support and uncertainty surrounding pregnancy and governmental funding/corporate sponsorship for elite pregnant athletes.^18^ Participants in the current study also highlighted significant internal and external barriers to melding pregnancy with elite sport, and they identified key support that would enable success with both.

The gender gap in sport participation is well established, and researchers have documented the various barriers to such participation, including elite athletes’ need to navigate the desire to become a parent with the desire to remain in elite sport. A variety of organisations across the world have emerged to advocate for increased participation of women in sport. At the Olympic level, the Women in Sport Commission was developed to advise the IOC and promote equal opportunities for women to participate in sport.^23^ Supporting and encouraging an elite athlete’s pathway to become pregnant and return to elite sport will encourage a more inclusive sporting environment. Athletes shared in-depth experiences which identified actionable steps that would be supportive of this endeavour. Specifically, athletes’ experiences of motherhood and elite sport were greatly influenced by the culture of the organisation. Athletes identified the critical need to have role models who became pregnant and returned to elite sport, as well as the need for a supportive network consisting of teammates, coaches and family. Without an open and supportive environment, many athletes were worried about disclosing their pregnancy out of fear of being viewed as undedicated to their sport, often delaying disclosure until late in pregnancy. Organisations with clear policies and regulations about the financial support and eligibility allowed athletes to make informed decisions on when and whether to get pregnant.

Many athletes highlighted the lack of research and guidelines around safe sport participation for elite athletes. The paucity of evidence available to them left athletes feeling vulnerable and unsure about whether they could or should participate in sport during pregnancy and degraded the trust they had in their healthcare providers who provided recommendations. The urgent need to better understand the implications of elite sport participation during pregnancy for maternal/fetal health was strongly evident.

These data provide important insight into the struggles of being both a mother and an elite athlete. Participants identified key action items that would facilitate future athletes in this endeavour (see table 2). Strengths of this study include the inclusion of elite athletes from a variety of team and individual sports from both summer and winter games. All athletes were pregnant in the last 5 years, reflecting recent experiences through in-depth interviews. Our interdisciplinary team of researchers included experts in qualitative and quantitative research, and included researchers, mothers, clinicians and former elite-level athletes to provide a well-rounded perspective. However, we also acknowledge some important limitations. The athletes who participated responded to recruitment advertisements on social media and/or word of mouth. As a result, most athletes were from North America and a few were from Europe. Pregnant athletes from other regions (eg, Asia and Africa) and low-income or middle-income countries likely have unique contextual experiences that should be explored in the future. Only athletes who identified as women participated in this study, and findings may not represent the complex experiences of pregnant trans men athletes. Although not studied, many issues may also be relevant to subelite and recreational athletes. Future research is required to better inform guidelines for pregnant athletes across the spectrum of competitiveness to achieve more inclusive, supportive and evidence-driven practices.

## Conclusion

The participants’ shared experiences highlight the numerous challenges of trying to navigate pregnancy alongside elite sport participation. Grounded in the voices of elite female athletes, findings from this research identify key themes and actionable steps for teams, national sport organisations, sponsors and international sporting bodies to support athletes during pregnancy. Creating clear policies and practices that support pregnant athletes is critical for ensuring equitable sport opportunities for female athletes.

**Key messages**

**What are the findings?**

- Research involving elite female athletes is necessary to support evidence-informed policies and practices regarding sport participation in pregnancy.
- Elite female athletes have to ‘make the tough decision’ between being a mother or an elite athlete in a system that typically does not support both. This study presents policy recommendations and key actionable steps to support female elite athletes to thrive as elite athlete mothers.

**How might it impact on clinical practice in the future?**

- Clinicians, coaches and sport organisations working with elite athletes who are pregnant or contemplating pregnancy should consciously create an environment and network that values and supports pregnancy. Research into elite sport participation during pregnancy should be prioritised by researchers, funding bodies and national/international sport organisations in consultation with clinicians and athletes to inform evidence-based clinical recommendations and guidance.

## Data Availability

All data relevant to the study are included in the article or uploaded as supplementary information. Not applicable.
