# Supplementary material for: Pushing for change: a qualitative study of the experiences of elite athletes during pregnancy
Source: Br J Sports Med. 2022 Feb 8;56(8):452–7. doi: 10.1136/bjsports-2021-104755 (PMC8995814; doi:10.1136/bjsports-2021-104755)
Supplement: Supplementary data [file bjsports-2021-104755supp001.pdf]

Methods

Study Design

Qualitative description was employed as the study design. Qualitative description, as described by Sandelowski (2000), is often used by researchers when there is a dearth of knowledge within a subject area, and when a detailed description is sought. Being less interpretive than other qualitative study designs, qualitative description supports researchers in staying "data-near" (Sandelowski, 2010). This process of staying near to the data facilitates simple or straightforward answers to questions that are relevant to practitioners and policy makers (Sandelowski, 2000), such as how elite female athletes navigate pregnancy. Qualitative description has been successfully applied in various other sport and physical activity-related studies, including some of our own research (e.g., Larson et al., 2018; McCormack et al., 2020). This study was approved by the University of Alberta Institutional Research Ethics Board (PRO00104326).

Participants

Between January and June 2021, we recruited elite female athletes through social media (i.e.,Twitter, Facebook, Instagram), and word of mouth via a purposeful and snowball sampling approach(20). In order to be eligible, athletes had to be $\geq$18 years old and have trained and/or competed at the highest level of their sport immediately prior to, and during pregnancy within the last five years (2016-2021). Prior to participation, individuals provided written, informed consent, and completed a brief questionnaire about their sporting background, demographics, and an overview of their pregnancy experiences. Specifically, the questionnaire included items such

as the highest level of competition achieved, the duration/type of training during pregnancy, and specifics of delivery (e.g., birth weight).

The experiences of 20 athletes from 11 Olympic (team and individual) sports are represented in this study. The majority of the participants were from North America with additional athletes from Europe. Participants were an average 35 ± 5 years of age with 15 of 20 currently training or competing at the elite level (see Table 1). To ensure anonymity of the participants, a complete list of the athletes' sports is not provided, and pseudonyms have been used in the reporting of the results.

Table 1: Participant Characteristics

Age (years)	35 ± 5
Retired	20% (n=4)
Elite sport participation (years)	8.8 ± 5.7
Type of sport	
Team	50% (n=10)
Individual	50% (n=10)
Highest level of competition	
Olympic Games or World Championships	50% (n=10)
National/International Competition	50% (n=10)

Data Generation

Qualitative description offers researchers the flexibility to use various forms of data generation and analysis; however, in-depth interviews are often used to explore the nature and shape of participants' personal experiences (Sandelowski, 2000). As such, once eligibility was confirmed, participants engaged in a one-on-one semi-structured interview; the interview guide was informed by sport and pregnancy-related literature and a Research Advisory Board (RAB). The RAB included researchers with expertise in qualitative and quantitative research; the all-female RAB also included researchers who are mothers, clinicians, and former elite level athletes.

The interview guide consisted of twelve major questions relating to athletes' experience (e.g., “Tell me about your experience becoming pregnant while training or competing at the elite level?”). Given the semi-structured nature of this interview guide, the interviewers (i.e., co-authors and RAB members AN and LR) had the flexibility to probe participants to elaborate on specifics provided in their responses. Both interviewers have expertise in qualitative research and research interests in psychosocial aspects of sport participation.

Interviews lasted an average of 60 minutes and took place between January 2021 and June 2021. Interviews took place during the Covid-19 pandemic; thus, all interviews were conducted via video-conferencing that were audio-recorded and transcribed verbatim by Otter.ai. This complied with the University's REB Covid-19 protocols and facilitated the team’s success in recruiting participants who were geographically dispersed across Canada and beyond. As well, immediately following each interview, a narrative summary of each interview was created by the interviewer and shared with members of the RAB.

Data Analysis

Researchers who employ a qualitative description study design prioritize staying close to the data and presenting a comprehensive summary of the findings. Therefore, content analysis is an ideal approach for analyzing the data generated from the interviews (Sandelowski, 2000). Elo and Kyngas' (2008) three phase approach (i.e., preparation, organization, and reporting) to content analysis was utilized in analyzing the findings of this research project. In the *preparation* phase, the verbatim transcripts were checked for accuracy and the unit of analysis was selected (i.e., the words of participants). To familiarize themselves with the data, the RAB read and re-

read the transcripts and narrative summaries. The *organization* phase involved a process of open-coding. AN and LR lead the initial process of "open coding" whereby they recorded notes in the margins of all transcripts. Other members of the RAB also engaged in a process of open coding to ensure their familiarity with the data as well. The RAB met weekly via Zoom to discuss the project. During such meetings, the team discussed similarities and differences between the codes they had identified. The data were classified, and re-classified, into comprehensive higher order themes until consensus was achieved among the RAB. Results were then shared with all participants, who were asked to provide any feedback or comments to be integrated into the reporting of findings. In terms of the *reporting* phase of analysis, direct quotes from participants were used to describe and support each theme.

Consistent with the constructivist epistemology that informed this research, a realist approach to validity was applied within this study. As described by Ronkainen and Wiltshire (2021), realist approaches to validity have largely been overlooked within the broad field of sport and exercise research, yet they facilitate critical thinking and practical techniques to reduce threats to validity. As such, consistent with the recommendations of Ronkainen and Wiltshire, our team employed numerous techniques to ensure descriptive validity, interpretative validity, and theoretical validity within this research. The three forms of validity were originally described by Maxwell (1992), yet conceptualized within the field of sport and exercise research by Ronkainen and Wiltshire (2021). To enhance descriptive validity, the transcripts of all interviews were checked for accuracy by members of the RAB and detailed notes and interview summaries were created for each interview. In terms of interpretative validity, multiple members of the RAB were involved in the data analysis process including coding and participants were provided an opportunity to comment on proposed results. Finally, in terms of theoretical validity, the research

findings highlight practical suggestions for policy and practice that can guide actions in the real world.

References

Larson, H. K., Mcfadden, K., McHugh, T. L. F., Berry, T. R., & Rodgers, W. M. (2018). When you don't get what you want—and it's really hard: Exploring motivational contributions to exercise dropout. *Psychology of Sport and Exercise*, *37*, 59-66.

Maxwell, J. (1992). Understanding and validity in qualitative research. *Harvard Educational Review*, *62*(3), 279-301.

McCormack, G. R., Nesdoly, A., Ghoneim, D., & McHugh, T. L. (2020). Realtors' perceptions of social and physical neighborhood characteristics associated with active living: A Canadian perspective. *International Journal of Environmental Research and Public Health*, *17*(23), 9150.

Ronkainen, N. J., & Wiltshire, G. (2021). Rethinking validity in qualitative sport and exercise psychology research: A realist perspective. *International Journal of Sport and Exercise Psychology*, *19*(1), 13-28.

Sandelowski, M. (2000). Whatever happened to qualitative description? *Research in Nursing & Health*, *23*(4), 334-340.

Sandelowski, M. (2010). What's in a name? Qualitative description revisited. *Research in Nursing & Health*, *33*(1), 77-84.

BMJ Publishing Group Limited (BMJ) disclaims all liability and responsibility arising from any reliance placed on this supplemental material which has been supplied by the author(s)

Semi-Structured Interview Guide

1. Tell me about your involvement in sport, particularly at the elite level.

2. What factors do you think *support* female athletes' participation in sport?

3. What factors do you think *limit* female athletes' participation in sport?

4. Tell me about your experience [or your athlete's experience] becoming pregnant while training or competing at the elite level?

5. What *supports* are in place in your sport with respect to athlete pregnancy?

6. What factors do you think *limit* elite athletes' right to pregnancy?

7. Tell me about your experience [or your athlete's experience] with return to sport at the elite level following delivery?

8. What *supports* are in place in your sport with respect to return to sport following delivery?

9. What factors do you think *limit* elite athletes' right to return to sport following delivery?

10. What factors must be addressed in policies or regulations to support elite athletes whom are pregnant?

11. What factors must be addressed in policies or regulations to support postpartum elite athletes and their return to sport?

12. What have we not discussed today, but you think is important to share with respect to the experiences of pregnant or postpartum elite athletes?

Participant Questionnaire

1) Current date
2) Date of birth
3) What is your ethnic background?
4) Where were you born?
5) What is your gender identity?

Your sporting career:

1) What sport(s) did you play at the elite level (elite is defined as training for or competing in national/international competitions at the highest level of competition, or the highest division in your sport).
2) How many years did you play your sport?
3) How many years were you playing at the elite level?
4) Please indicate the highest level you trained or competed at (for example, I was a speed swimmer at the 2012 Olympic games, I played soccer for Canada's National Team, I was a carded athlete etc).
5) Did you return to elite level sport in the postpartum period?
    a. When did you return to elite level training or competition?
    b. If not, why not?
6) Are you retired from elite level sport?
7) Why did you retire from elite level sport?
